# A cross-sectional survey: knowledge, attitudes, and practices of self-medication in medical and pharmacy students

**DOI:** 10.1186/s12913-022-07704-0

**Published:** 2022-03-17

**Authors:** Razan Khalid Alduraibi, Waleed Mohammad Altowayan

**Affiliations:** 1 College of Medicine, Qassim University, Box 3499, Buraydah – 669, Qassim, 52385 Saudi Arabia; 2 Department of Pharmacy Practice, College of Pharmacy, Qassim University, Qassim, Saudi Arabia

**Keywords:** Knowledge, Self-medication, Medical student, Qassim university

## Abstract

**Background:**

Self-Medication is common practice worldwide in both developed and developing countries. Self-Medication is referred as self consumption of medication without consulting a physician for either diagnosis or treatment. This study aimed to assess the knowledge, attitudes and practices toward self-medication among medical and pharmacy students.

**Methods:**

This cross-sectional study was conducted among medical and pharmacy students in Qassim university, Buraydah, Saudi Arabia, during the period 2020–2021. Multistage random sampling technique was used to recruit students. The data were collected through questionnaire.

**Results:**

Three hundred and sixteen of 316 students were recruited. This study showed that the majority (94.6%) of students had good knowledge of self-medication. Additionally, the following characteristics were significantly associated with good knowledge: being female, and Pharmacy students. Overall mean score for the attitudes towards self-medication shows that 58.4% of the total sample had high agreements towards the questions of the attitudes toward self-medication. More than half (63.9%) of the students reported that they practice self- medication in the last 6 months. Pain killers was the most common medication used for self- medication by the majority of the students (88.29%).

**Conclusions:**

In conclusion, students’ knowledge of self-medication appears to be good and significantly high among pharmacy students in comparison to medical students. As well self-medication was highly practiced among the students. Therefor, medical and pharmacy students should be viewed as important contributors to the public health care system, and future health professionals should be properly educated on good pharmacy practice and responsible self-medication.

**Trial registration:**

Not applicable.

## Background

Self-medication (SM) is a common practice in both developed and developing countries worldwide. SM is referred to as self-consumption of medication without consulting a physician for either diagnosis or treatment. The World Health Organization (WHO) defines SM practice as “use of over-the-counter medication (OTC) to treat self-diagnosed symptoms or disorders, or for the continuation and reuse of prescribed medications for recurrent diseases” [1]. SM has various forms, including taking medications without a physician’s prescription, using a previous prescription for a similar condition, or using drugs obtainable at home without getting a physician’s advice. SM can be a serious issue and can lead to several problems, such as negative pharmaceutical reactions, a possible increase in antimicrobial resistance, and can be a waste of resources [2].

The most prominent causes encouraging SM in Saudi Arabia and across the world are previous disease experience, insufficient information about diseases, economic problems, insufficient time to visit a physician, and easy access to drugs [3, 4].

SM was found to be on the rise in prior surveys released from various countries. For example, in Bangladesh, Jordan, Egypt and Ethiopia, the prevalence of SM was 88, 86.7, 52.7, 32.7, and 70.8%, respectively [5–9].

In Saudi Arabia, high prevalence percentages of 98.7, 86.6%, 81,69, 64.8, and 19.61% have been found in a few studies that have been conducted to report SM prevalence among university students [3, 10–13]. Two of these studies were carried out at Qassim University [10, 13].

Medical and pharmacy students are generally different from other university students, as they are exposed to knowledge about diseases and drugs, which makes SM easier for them to practice. Therefore, pharmacists and physicians have an important role in providing useful recommendations on the proper and safe usage of pharmaceutical drugs. To the best of our knowledge, no study has focused on investigating the knowledge, attitudes, and practices of medical and pharmacy students toward SM in the Qassim region. Therefore, our objective was to assess the knowledge, attitude, and SM practices of medical and pharmacy students at Qassim University and to examine the association between demographic characteristics, knowledge and attitudes.

## Methods

### Study setting

A cross-sectional study was conducted using a questionnaire among medical and pharmacy students in Qassim University, Buraydah, Saudi Arabia, during the 2020–2021 period.

A multistage random sampling technique was used to choose the respective number of students. Medical and Pharmacy Colleges, plus entry years were considered strata. Students were randomly selected from each stratum. All medical and pharmacy students from year one to year four were included. Medical and pharmacy students were excluded if they were not available during the study period or refused to participate.

### Sample size

The sample size was calculated by using a standard sample size equation “n = z2p(1-p)/e2” and a proportion of 50% (the assumed proportion of students who had good knowledge of and attitudes toward SM). Using a 95% confidence interval and a 5% margin of error, the sample size was estimated to be 285 and was adjusted to 342 to compensate for the nonresponse rate.

### Validity and reliability

The questionnaire was developed by the principal investigator based on the study objectives and after a literature review of similar studies. A panel of 3 physicians, all of whom provided clinical care for patients familiar with the survey’s development, assessed the questionnaire for appropriateness, accuracy, and relevance, plus were asked to critique the questionnaire’s content. To ensure the face validity of the questionnaire, it was presented to a sample of 20 students on two occasions. These students were chosen from medical and pharmacy faculties and were from different academic years. The results of the piloted questionnaires were not included in the analysis. To estimate the internal consistency of the items, a reliability scale evaluation was performed. A good Cronbach’s alpha (α = 0.75) score was achieved.

As the research was conducted during the COVID-19 pandemic, students were asked to join a Zoom meeting conducted by the investigators to enforce social distancing. The aims of the study were explained to the students, and they were asked for consent. A standard pretested questionnaire was prepared using Google forms and emailed to 342 students after receiving their consent. Two reminder emails were sent after an interval of 1 month.

The questionnaire was divided into four sections with a total of 33 questions. The first section pertained to demographic characteristics, including age, gender, level of education, and nationality. The second section assessed the students’ knowledge regarding SM. The response choices for knowledge items included “yes”, “no” and “do not know”. Correct answers were scored as 1, while incorrect answers and “do not know” were scored as 0. The total knowledge score ranged from 0 to 7 (7 items). Knowledge was defined as poor for a score of 0–4 and good for a score of 5–7.

The third section assessed the attitudes of students toward SM. Five-point Likert scale items were used; strongly agree responses were scored as 5, agree as 4, uncertain as 3, disagree as 2, and strongly disagree as 1.The fourth section assessed the SM practices of students. Questions were asked about the types of drugs used and ailments experienced by the students, as well as their reasons for and the negative outcomes of self-medication.

### Data management and analysis plan

The data collected through Google Form were recoded and entered using the Statistical Package SPSS (v.26), to describe the basic features of the data in the study through frequencies and percentages. Spearman’s rho test was used to test the relation between knowledge and attitudes toward SM, since both were assessed using ordinal values. Chi-squared test (χ2) was used to find the association between the level of knowledge (poor - good) and sociodemographic variables. Finally, ANOVA was used to find the differences in the mean attitude scores of the 5-Likert scale according to sociodemographic variables.

### Ethical approval

Approval for the study was obtained from the Institutional Review Board, College of Medicine, Qassim University (no. 20–07-03), Qassim, Saudi Arabia. Each participant received the questionnaire and was informed about the objective of the present study. All participants provided an informed google consent form. The Institutional Review Board has agreed that completing the questionnaire will imply consent.

## Results

### Demographic information

Three hundred and sixteen of the 343, students completed the questionnaires (response rate of 92%).

Table 1 shows that the total sample size was 316; 54.4% were female, and 45.6% were male. The most common age group was 21–23 years, accounting for 68.7% of the total sample, while 83.5% lived in urban areas and 16.5% lived in rural areas. Regarding specialty, 50.3% studied medicine and 49.7% pharmacy. Regarding education level, the highest percentage was 34.5% for first year students. It was seen that 86.1% did not have health insurance, and 88.3% reported having no medical illness.

**Table 1:** Demographic characteristics of the students (*n* = 316)

	Groups	*N*	%
Gender	Male	144	45.6
	Female	172	54.4
Age	18–20	60	19.0
	21–23	217	68.7
	24–27	39	12.3
Area of living	Urban	264	83.5
	Rural	52	16.5
Specialty	Medicine	159	50.3
	Pharmacy	157	49.7
Education level	First year	109	34.5
	Second year	52	16.5
	Third year	83	26.3
	Fourth year	72	22.8
Do you have health insurance?	Yes	44	13.9
	No	272	86.1
Do you have any medical illness?	Yes	37	11.7
	No	279	88.3

### Students’ knowledge of self-medication

**Fig. 1:**
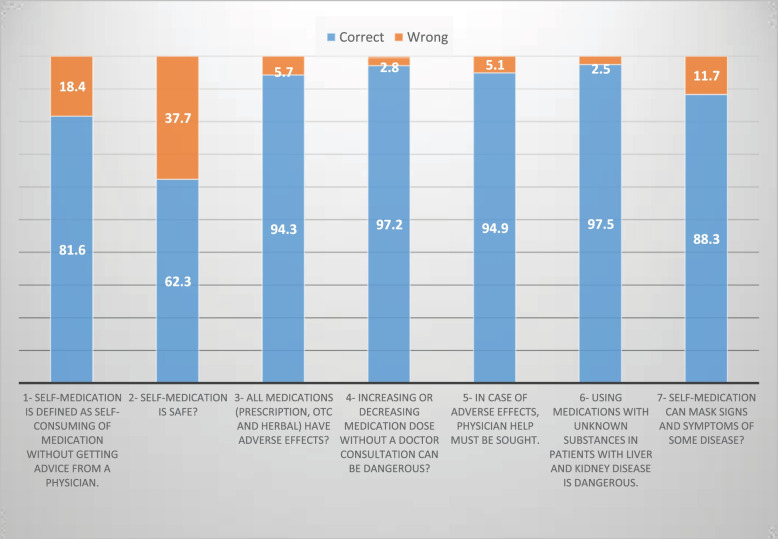
Self-medication knowledge of Medical and Pharmacy students

More than half (81.6%) of students had adequate knowledge that SM is defined as self-consuming medication without receiving advice from a physician. Moreover, the majority (94.3%) of students knew that all medications (prescription, OTC and herbal) had adverse effects. Most of them (94.9%) recognized the importance of seeking physician help in case of adverse drug effects. A total of 308 (97.5%) students were aware that using medications with unknown substances in patients with liver and kidney disease is dangerous, and nearly all students knew that increasing or decreasing medication doses without a doctor consultation can be dangerous. More than half (88.3%) of students knew that SM can mask signs and symptoms of some diseases (Fig. 1).

The total knowledge scores ranged between 0 and 7; a poor level was considered for a total score of 0, 1, 2, 3 or 4 out of 7, and a good level was considered for a total score of 5, 6 and 7 out of 7. The results show that 17 out of 316 students, which represent 5.4% of the total sample, had poor knowledge of SM, indicating total scores lower than 5, while the other 299 students, which represent 94.6% of the sample, had good knowledge of SM, indicating total scores of 5 and above.

Table 2 demonstrates a statistically significant association (*P* < .05) between good knowledge and the following student characteristics: being female and pharmacy students.

**Table 2:** Association between knowledge and demographic variables (*n* = 316)

		Knowledge Level		Chi-Square	*p* value
		Poor	Good		
Gender	Male	8.3%	91.7%	4.534	0.033*
	Female	2.9%	97.1%		
Age	18–20	5.0%	95.0%	.468	0.791
	21–23	5.1%	94.9%		
	24–27	7.7%	92.3%		
Area of living	Urban	6.1%	93.9%	1.461	0.227
	Rural	1.9%	98.1%		
What is your specialty?	Medicine	9.4%	90.6%	10.334	0.001**
	Pharmacy	1.3%	98.7%		
Education level	First year	8.3%	91.7%	7.069	0.070
	Second year	1.9%	98.1%		
	Third year	1.2%	98.8%		
	Fourth year	8.3%	91.7%		
Do you have health insurance?	Yes	9.1%	90.9%	1.383	0.240
	No	4.8%	95.2%		
Do you have any medical illness?	Yes	2.7%	97.3%	.590	0.442
	No	5.7%	94.3%		

*significant at *p* < .05

### Students’ attitudes toward self-medication

**Fig. 2:**
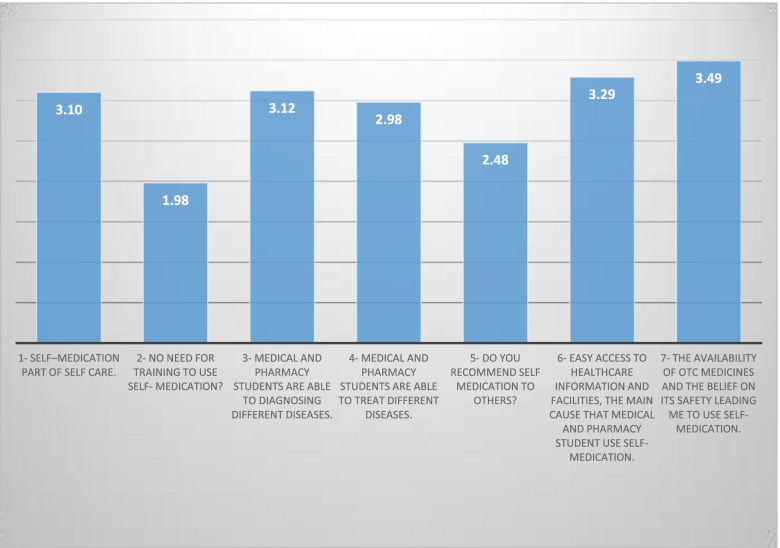
Attitudes of medical and pharmacy students toward self-medication in Al-Qassim university

Figure 2 shows the students’ responses to the seven questions on attitudes toward SM. The highest mean score was 3.49 out of the 5-point scale for (The availability of OTC medicines and the belief on its safety leading me to use SM), followed by mean score 3.29 for (Easy access to health care information and facilities, the main cause that medical and pharmacy student use self- medication). The lowest mean score of agreement was 1.98 out of 5 (no need for training to use SM?). The overall mean score for attitudes toward SM was 2.92 out of the 5 scales, with an SD of 0.692, which represents approximately 58.4% of the total sample, and has high agreement with the questions on attitudes toward SM at Qassim University, Saudi Arabia.

Table 3 demonstrates a statistically significant difference (*P* < .05) in the mean attitude score and the following student characteristics: being male, being 21–23 years old, and being pharmacy students.

**Table 3:** Association between knowledge score and attitudes score (*n* = 316)

	Spearman’s rho	*P* value	Significance	
1- Self–medication part of self care.	−0.152	0.007	< 0.001	S
2- No need for training to use self- medication?	−0.087	0.123	> 0.05	N.S.
3- Medical and pharmacy students are able to diagnose different diseases.	−0.063	0.264	> 0.05	N.S.
4- Medical and pharmacy students are able to treat different diseases.	−0.089	0.115	> 0.05	N.S.
5- Do you recommend self medication to others?	−0.134	0.017	< 0.05	S
6- Easy access to health care information and facilities, the main cause that medical and pharmacy student use self- medication.	0.129	0.022	< 0.05	S
7- The availability of OTC medicines and belief of its safety leading me to use SM.	0.082	0.148	> 0.05	N.S.
Attitude	−0.093	0.097	< 0.10	S

A statistically significant negative relation between knowledge and the self–medication part of self-care (*r* = − 0.152, *p* <  0.05). The higher the knowledge of SM, the lower the believe in the SM part of self care. In addition, there was a statistically significant negative relation between knowledge and recommendation of SM to others (*r* = − 0.143, *p* <  0.05). Hence, the higher the good knowledge of SM, the lower the recommendation of SM to others.

### Students’ practices toward self-medication

The students’ practice towards the statements about SM is illustrated in Table 4. More than half (63.9%) of the students reported that they had practiced SM in the last 6 months. The majority (79.1%) of respondents knew the medication classification of OTCs and prescription drugs. Pain killers were the most common medication used for SM by the majority of the students (88.29%), followed by antipyretics (49.68%). It was also observed that 36.71% of the students reported having self-medicated with antihistamine; others are listed in Table 4.

**Table 4:** Students’ practices toward self-medication (*n* = 316)

Question		*n*	%
1- Did you practice SM in the last 6 months?	Yes	202	63.9
	No	114	36.1
2- How frequently did you visit the pharmacy to purchase drugs without a prescription for yourself in the last 6 Months?	Once	153	48.4
	Twice	65	20.6
	Three times	60	19.0
	Four times	16	5.1
	Five times	3	0.9
	More than five times	19	6.0
3- Do you know if the medicines you consumed needed prescription or not?	Yes	250	79.1
	No	33	10.4
	I don’t know	33	10.4
4- Which of the following drugs have you taken without prescription during the last 6 months?	Pain killers	279	88.29
	Antibiotics	110	34.81
	antipyretics	157	49.68
	Antihistamines	116	36.71
	Cough syrups	42	13.29
	Cold and flu preparations	59	18.67
	Antacid drugs	30	9.49
	Drugs for constipation	42	13.29
	Drugs for diarrhea	9	2.85
	Anti-emetics	12	3.8
	Nasal/Ear/Eye drops	100	31.65
	Topical agents (skin treatment	96	30.38
	Nutritional/energy supplements	92	29.11
	Herbs	69	21.84
5- For which of the following indications have you taken medications without prescription during the last 6 months?	I do not take	199	62.97
	Headache	204	64.56
	Cough & common cold	110	34.81
	Fever	98	31.01
	Infection	22	6.96
	Heart burn	23	7.28
	Allergy	47	14.87
	Disorder of digestive system	37	11.71
	Body pain	66	20.89
	Tooth pain	41	12.97
	Acne/skin diseases	90	28.48
	Menstrual problems	78	24.68
	Insomnia	22	6.96
6- Source of Information About SM	Relatives	68	21.52
	Friends	32	10.13
	Personal knowledge	205	64.87
	Multi media	84	26.58
	Advised by Doctors but without prescription	114	36.08
	Pharmacists or those working in the pharmacy	160	50.63
7- What do you know about the drug you requested?	Name of the drug	275	87.03
	Indication	237	75
	Dose	216	68.35
	How to use	246	77.85
	Frequency	200	63.29
	Duration	183	57.91
	Storage of the drug at home	158	50
8- Do you know the potential adverse reactions of the drug with which you self-medicated?	Yes	219	69.3
	No	57	18.0
	I don’t know	40	12.7
9- From where did you SM?	Pharmacy	298	94.3
	Street market	14	4.43
	Herbal store	38	12.03
	Relative/friend	28	8.86
10- Reasons for SM	To save money	49	15.51
	To save time	145	45.89
	Privacy	33	10.44
	Needed quick relief	201	63.61
	No hospital nearby	36	11.39
	Previous experience	158	50
	Health problem not serious	212	67.09
	Embarrassed of discussing own symptoms	8	2.53
11- Have you ever experienced a negative side effect after SM?	Yes	71	22.5
	No	245	77.5
If yes	Drug side effects	49	69.01
	Disease recurrence	12	16.9
	Resistance to drug	9	12.68
	Drug interactions	8	11.27
12- Do you feel confident with the use of SM?	Yes	246	77.8
	No	70	22.2

More than half (64.56%) of respondents reported that the most frequent causes for the practice SM were headache followed by cough and the common cold (34.8%). Other episodes included fever, acne, menstrual problems, body pain and allergies (31, 28.48, 24.68, 20.89, and 14.87%, respectively). More than half (64.87%) of respondents reported that their personal knowledge was the major source of information for the practice of SM. Approximately two-thirds (69.3%) of respondents knew the potential adverse reactions of the drug. The majority (94.3%) of respondents reported that they obtained SM from a pharmacy, and 12% used SM from herbal stores.

Two-thirds (67%) of respondents reported that they self-medicated because of non seriousness of the illness, followed by quick relief (63.61%). Approximately one-quarter (22.5%) of respondents reported that they experienced a negative side effect after SM. Approximately 69% of them reported side effects from the drug. The majority (77.8%) of respondents reported that they felt confident about the use of SM.

## Discussion

To our knowledge, this study is the first to assess the knowledge, attitudes and practices of medical and pharmacy students regarding SM at Qassim University. It has demonstrated essential findings. First, this study showed that the majority (94.6%) of the students had good knowledge scores. Second, more than half (58.4%) of students had agreeable attitudes toward SM. Third, more than half (63.9%) of the students had practiced SM in the last 6 months.

SM for minor diseases and non serious health conditions appears to be a common practice among students. In Saudi Arabia and other countries, the prevalence of SM practice varies widely throughout all demographic groups. A study by Alshahrani reported the highest prevalence of SM practice in Saudi Arabia, whereas Makeen et al., reported the lowest frequency (98.7 and 11.4%, respectively) [3, 14]. These findings are similar to another study conducted in Saudi Arabia, which found that 64.8% of medical students practice SM [11]. Variations in study sample demographics, research methodology, data collection methodologies, and variation in response rates across studies may have resulted in a wide range of prevalence across different regions.

SM appears to be more prevalent among students than in the general population. One study on Ethiopian medical and pharmacy students found a 38.5% prevalence [9]. SM was also reported by 44.8% of Bahraini medical students [15], 78.6% of Indian medical students [16], and 55.2% of Egyptian medical students [17]. SM was reported by 98% of Palestinian students in another survey [18]. Two European studies, one on Slovenian students and the other on Serbian students, found frequencies of 92.3 and 79.9%, respectively [19, 20]. This could be attributable to a variety of factors, including students’ greater pharmacological and clinical educations, or better internet access.

Regarding knowledge, 94.6% of our students had a good level of knowledge about SM. The ratio of pharmacy students who had good knowledge on this issue was significantly higher than that of medical students. This observation is most likely due to differences in educational courses and curricula within these fields. Furthermore, female students had significantly higher knowledge than male students. This finding is similar to a study conducted among pharmacy students in Iran [21].

With regard to the practice of SM, our results showed that 63.9% of the pharmacy and medical students of Qassim University who participated in the study had at least one instance of SM during the past 6 months. Adequate knowledge and the non acute nature of the condition were identified to be the most important reasons for SM. Likewise, two studies conducted in China and Brazil concluded that the most common reason for SM was a non serious or transient disease (45 and 46%, respectively) [22, 23]. In a Rwandan study, having a non serious disease was the most common reason for SM among university students [24]. Financial and insurance related issues have been reported as other reasons for SM [25].Another study found that bad behavior by health care providers, being too far away from a clinic, and low efficacy of prescribed drugs were all used as justifications by university students for SM [26].

The most widely utilized medications for SM among our students were painkillers and antipyretics (60%). Similarly, our findings were in line with the findings of the majority of similar studies. Painkillers (60%) and antipyretics were the most commonly used drugs for SM among our students. Likewise, our results were consistent with the findings of most similar studies [17, 27, 28]. In other studies, NSAIDs [23, 29], antibiotics [23, 24, 26], and pain killers [26, 29] were reported as the most common drugs used for SM. In a study conducted in Pakistan, OTC drugs were found to be the most commonly used pharmaceuticals (98.3%) by medical undergraduate students [30]. The primary reason for using analgesics and antipyretics for SM is that they do not require a medical prescription, plus are easily and quickly available in pharmacies and other general stores nearby. Our study also revealed that very few students anticipated adverse drug reactions, indicating that all students, regardless of study level, have at least basic knowledge of the drugs used for SM.

A large number of students’ attitudes toward SM agreed that SM is an important part of self-care. As a result, students should be aware of the dangers of improper medication use, which can lead to drug resistance, toxicity, and increased adverse effects. Despite the fact that the majority of students use SM, they do not recommend it to others. This attitude explains why most students’ perceptions of SM change as they gain knowledge during their studies.

A limitation of this study is the use of a survey tool that has not undergone prior reliability and validity testing. In addition, the results of this study cannot be generalized to other populations in the country because knowledge, attitude, and practice (KAP) might be greatly influenced by sociodemographic factors in the population. More studies on SM need to be performed, especially with increasing SM practice in the region.

## Conclusions

In conclusion, medical and pharmacy students’ knowledge of SM appears to be good and is significantly higher among pharmacy students than among medical students; SM was highly practiced among these students. Therefore, medical and pharmacy students should be viewed as important contributors to the public health care system, and as future health professionals, they should be properly educated on good pharmacy practices and responsible SM.

## Data Availability

The datasets used and/or analyzed during the current study are available from the corresponding author on reasonable request.

## Abbreviations

SM: Self-Medication
KAP: Knowledge, attitude, and practice
WHO: World Health Organization
OTC: Over-the-counter medication
